# The Mediating Role of Organizational Reputation and Trust in the Intention to Use Wearable Health Devices: Cross-Country Study

**DOI:** 10.2196/16721

**Published:** 2020-06-09

**Authors:** Funmi Adebesin, Revingstone Mwalugha

**Affiliations:** 1 Department of Informatics University of Pretoria Pretoria South Africa

**Keywords:** fitness trackers, intention, Kenya, physical activity, privacy, South Africa, trust, regression analysis

## Abstract

**Background:**

The use of consumer wearable health devices for fitness tracking has seen an upward trend across the globe. Previous studies have shown that trust is an important factor in the adoption and use of new technologies. However, little is known about the influence of organizational reputation and trust on the intention to use wearable health devices.

**Objective:**

This study aimed to investigate the mediating role of organizational reputation and trust in the intention to use wearable health devices and to examine the extent to which the country of residence influenced the effect of organizational reputation on consumers’ trust in and intention to use wearable health devices.

**Methods:**

We conducted a cross-country survey with participants from Kenya and South Africa using a Google Forms questionnaire derived from previously validated items. A series of mediation regression analyses were carried out using the PROCESS macro with the bootstrap CI procedure. A one-way, between-group multivariate analysis of variance (MANOVA) was also used to determine the key factors that distinguish Kenyans and South Africans in their intention to use wearable health devices.

**Results:**

A total of 232 questionnaire responses were collected. The results revealed that organizational reputation significantly mediates the relationship between trust propensity and trust, with an indirect effect of 0.22 (95% CI 0.143-0.309). Organizational reputation also plays a significant direct role in the intention to use a wearable health device, with a direct effect of 0.32 (95% CI 0.175-0.483). This role is regardless of participants’ country of residence. Furthermore, there is a significant mediating effect of trust on the relationship between trust propensity and the intention to use a wearable health device, with an indirect effect of 0.26 (95% CI 0.172-0.349); between perceived security and the intention to use a wearable health device, with an indirect effect of 0.36 (95% CI 0.255-0.461); and between perceived privacy and the intention to use a wearable health device, with an indirect effect of 0.42 (95% CI 0.282-0.557). The MANOVA test shows statistically significant differences in all variables for both groups, with the exception of organizational reputation where there is no significant difference between the two cohorts.

**Conclusions:**

Organizational reputation has a significant direct influence on participants’ trust in and the intention to use a wearable health device irrespective of their country of residence. Even in the presence of perceived security and perceived privacy, trust has a significant mediating effect on the intention to use a wearable health device.

## Introduction

### Background

Wearable health technologies continue to rank among the top 3 global fitness trends since 2016, maintaining the first position in 2016, 2017, and 2019 [1]. There is growing evidence that increasing physical activity (PA) is beneficial to personal well-being [2-5]. Several studies reported on the positive relationship between health consciousness and increase in PA [6-8]. Wearable health devices that monitor PA can take the form of an accelerometer, a pedometer, a heart rate monitor, or a combination of these mechanisms [9-12]. As a result of their capability to seamlessly monitor and capture health data [13], wearable health devices have become popular and valuable tools for monitoring and recording PA and other health-related data [10,14,15].

Consumer wearable health devices (CWHDs), such as Apple Watch, Fitbit, Garmin, and Huawei, typically come with a range of features that support real-time tracking of PA. These devices combine various behavioral change techniques, including goal setting, self-monitoring, feedback, and reward [10], to promote positive health habits. In many instances, a wearable health device is linked to one or more compatible mobile phone apps. CWHDs have the potential to help users improve their PA and adopt healthy behavior [10,16]. Through features such as goal setting, performance monitoring, and personalized feedback, users can set up fitness goals that meet their needs, track activities against set goals, and adjust targets as required [16,17]. The virtual fitness trainer feature in CWHDs and mobile fitness apps can mimic a personal fitness coach or trainer by showing users the correct body movements for a specific exercise [18,19]. This feature is particularly beneficial in the Global South context (countries classified as low- and medium-income countries in Africa, Asia, Latin America, and the Caribbean), where many citizens do not have the financial means to use the services of a professional fitness trainer.

Despite the potential benefits of CWHDs in supporting increased PA, there are legitimate concerns over privacy and the security of data collected by these devices [20,21]. Data collected by CWHDs are often transmitted to cloud storage. These data are at risk of unauthorized access by people with malicious intent on the device itself, while the data are in transit and (or) on cloud storage [22].

Several studies have been published on the use of CWHDs to track PA [23-26]. However, as acknowledged by Wiesner et al [21], few studies have focused on the privacy issues that influence the adoption of these devices. Even rarer are studies by researchers from Global South countries focusing on the privacy and security of CWHDs. To address this gap, we investigated the mediating role of 2 variables (ie, organizational reputation and trust) in the following relationships:

- The mediating role of organizational reputation in the relationship between trust propensity and trust.
- The mediating effect of trust in the relationship between trust propensity, perceived security, and perceived privacy in the intention to use wearable health devices.
- The mediating role of trust in the relationship between organizational reputation and the intention to use a wearable health device.

The following research questions are addressed in the paper:

- To what extent is the relationship between organizational reputation and the intention to use a wearable health device mediated by trust?
- To what extent are the relationships between the 3 factors (ie, trust propensity, perceived security, and perceived privacy) and the intention to use a wearable health device mediated by trust?
- What are the key factors that distinguish Kenyans and South Africans in their intention to use wearable health devices?

Previous studies, such as Gao et al [27], Gu et al [28], and Meyer et al [29], on the factors that influence the adoption and use of wearable health devices did not consider the mediating role of factors such as organizational reputation. Furthermore, studies such as Sillence et al [30] that specifically investigated the influence of organizational reputation on women’s trust in a website were not from the perspective of mediation. Another unique feature of our study is the fact that it is a cross-country study, which allowed us to collect data from 2 developing countries that are culturally diverse, yet with stark similarities [31,32]. Hence, this paper contributes to the field of ubiquitous health (uHealth) as it presents empirical research findings on the effect of organizational reputation on consumers’ trust in wearable health devices and the intention to use wearable health devices. In the following subsections, we discuss the theoretical background upon which the constructs that were tested in the study are based.

### Trust Propensity and Trust

Trust propensity relates to an individual’s natural predisposition to trust another person. This trust is without regard for the circumstances, even with little or no previous information about the other person (or thing) [33,34]. A person (trustor) is said to be trusting of another party (trustee) when the trustor is prepared to be subjected to the consequences of the trustee’s actions. This willingness is based on the expectation that the trustee will behave as expected by the trustor regardless of whether the trustor has power to control the actions of the trustee [33]. Trust consists of 4 components, namely (1) *competence*, which is the belief that the other party is capable of discharging their responsibility to the trustor; (2) *benevolence*, the belief that the trustee is willing to act in the best interest of the trustor; (3) *integrity*, the belief that the trustee will be truthful, abide by agreements, act ethically, and fulfill promises made; and (4) *predictability*, the belief that over time, the actions of the other party will be consistent and the trustor can predict the trustee’s action in any given situation [35]. Trust propensity can influence the development of trust in a person or thing. Cultural background, developmental experiences, and personality types are thought to influence the willingness of an individual to trust other people or things [33].

Earlier studies have shown that trust propensity can shape consumers’ trust in the use of new technologies. For example, Zhou [36] found that trust propensity has a significant effect on initial trust in and the intention to use mobile banking apps. In her study on the role of trust in citizens’ adoption and use of electronic government (eGovernment), Colesca [37] reported a positive relationship between trust propensity and trust in eGovernment, whereas Lee and Turban [38] also reported that trust propensity has a positive influence on consumers’ trust in electronic commerce (eCommerce) websites. Similarly, Gu et al [28] reported a positive link between trust propensity and initial trust in CWHDs.

Although trust is an important factor in the adoption and use of technologies such as mobile banking and eCommerce websites, it is even more the case when it comes to CWHDs that collect and analyze health data on a continuous basis [14]. Design issues such as accuracy, privacy, and security of health data can influence the level of trust and eventual intention to use technologies such as CWHDs [29,39].

Although previous studies, such as those by Gu et al [28], Zhou [36], Colesca [37], Lee and Turban [38], and Ribadu and Rahman [40], have shown the link between trust propensity and trust, little is known about the mediating role of organizational reputation in this relationship. To address this gap, we hypothesized the following:

- *H1:* The relationship between trust propensity and trust is mediated by organizational reputation.
- *H2:* The relationship between trust propensity and the intention to use a wearable health device is mediated by trust.

### Organizational Reputation and Trust

Organizational reputation can be viewed from 2 perspectives, namely institutional and economic. From an institutional perspective, organizational reputation relates to the extent to which an organization creates value for its stakeholders and differentiates itself from its competitors [41,42]. From an economic perspective, organizational reputation is the ability of a company to produce quality goods and services [42]. Positive organizational reputation is generally seen as an intangible asset that can enable a company to grow its market share, maximize its profit, and attract new customers, while retaining existing ones [43-45].

Positive organizational reputation reflects the quality and performance of the products and (or) services being offered by an organization [46]. There is a direct link between organizational reputation and consumers’ trust and continued loyalty [41,47-52]. Although good organizational reputation is vital for building consumers’ trust in general, it is even more so in web-based services where customers are unable to feel or try out a product before making payments. Thus, customers may only have to rely on their *gut feeling* or the company’s reputation to inform the decision to interact with the web-based service [47,48].

Studies by Haery et al [47] and Jung and Seock [50] showed that positive organizational reputation influenced customers’ intention and continued loyalty. Flavián et al [52] also reported that organizational reputation has a significant effect on customers’ trust in web-based banking, compared with traditional banking methods. In a study that investigated the factors that influence women’s trust and mistrust in health websites, Sillence et al [30] also found that the majority of study participants were more trusting of information on health websites that is managed by reputable organizations. Thus, we can extend this relationship to consumers’ intention to use wearable health devices through the following hypothesis:

- *H3:* The relationship between organizational reputation and the intention to use a wearable health device is mediated by trust.

### Perceived Security and Trust

Perceived security can be defined as the belief that one’s personal information will not be accessed, viewed, manipulated, or stored by unauthorized persons while in transit or on a storage device (location) [53]. The mechanisms that can be used to ensure the security of customers’ information include digital signatures, encryption, authentication, and verification [54-57].

The impact of perceived security on trust in new technologies has been studied by several researchers. For example, in their study on the adoption of internet banking, Patel and Patel [58] found that perceived security has a strong influence on participants’ intention to use internet banking services. Similarly, Aboobucker and Bao [59] found that security and privacy play a significant role in the adoption of internet banking. In another study on the acceptance of near-field communication (NFC)–based mobile payment by restaurant users, Khalilzadeh et al [60] found that perceived security influences customers’ trust in NFC-based mobile payment systems. In another study, Sharma et al [61] found that perceived security and perceived privacy have a significant influence on the trust in and subsequent use of social networks.

Authors of previous studies have reported on privacy and security concerns related to uHealth data [62-64], which is arguably more personal than biographical data such as name, date of birth, or telephone number. However, little is known about the mediating role of trust in the relationship between perceived security and the intention to use a wearable health device. Data collected by wearable health devices are at risk of unauthorized access by people with malicious intent on the device itself, while the data are in transit and (or) on cloud storage [22,65,66]. To test the mediating role of trust in the relationship between perceived security and the intention to use a wearable health device, we formulated the following hypothesis:

- *H4:* The relationship between perceived security and the intention to use a wearable health device is mediated by trust.

### Perceived Privacy and Trust

Privacy can be defined as the right to determine or control what personal information is disclosed to other entities or third parties [67]. Concerns over the privacy of personal information are growing due to the increasing capability of new technologies to collect, process, and distribute large amounts of data [53]. Security measures provide technical assurance that, to keep personal data safe, best practices will be adhered to. Privacy, on the other hand, involves compliance with legal requirements and policies on how consumers’ personal data will be collected and used [53]. When asked to provide personal information on electronic platforms, a user will typically do a quick risk-benefit analysis. When the benefits are perceived to outweigh the associated privacy risks, the user is more likely to provide the requested information [27].

In their study on the acceptance of wearable technology in health care, Gao et al [27] found that one of the factors that influence the intention to adopt wearable health device for fitness tracking is perceived privacy risk. A high level of perceived privacy risk can have a negative influence on the trust in and intention to use new technologies. Conversely, a low level of perceived privacy risk will have a positive influence on consumers’ trust in and intention to use new technologies [28,61]. To determine the mediating role of trust in the relationship between perceived privacy and the intention to use a wearable health device, we proposed the following hypothesis:

- *H5:* The relationship between perceived privacy and the intention to use a wearable health device is mediated by trust.

We collected data from participants residing in 2 different countries, Kenya and South Africa. We cannot assume that participants from these countries are homogeneous, and the factors discussed in the preceding subsections would be applicable to the participants in the same way. Even for participants in the same country, this kind of an assumption would not be plausible. To investigate the key factors that distinguish participants from the 2 countries in their intention to use wearable health devices, we hypothesized the following:

- *H6:* There is a significant difference between Kenyans and South Africans in their intention to use wearable health devices.

## Methods

### Research Model and Measurements

The conceptual model of the relationships between trust propensity, organizational reputation, trust, perceived security, perceived privacy, and intention to use wearable health devices is illustrated in Multimedia Appendix 1. The questionnaire used in the study was based on constructs that have been validated and tested for their reliability by researchers as reflected in Multimedia Appendix 2. Items on the questionnaire were adapted to suit the purpose of our study through minor changes to their wordings. The items were measured using a 5-point Likert scale (1=strongly disagree to 5=strongly agree). The questionnaire went through a pretest process where 2 experts (one with experience in the field of electronic health and the other an expert in the design of quantitative studies) reviewed and made suggestions for its improvement. Following the modifications, a pilot study was conducted with 5 participants to test the usability of the questionnaire and determine if further modification is required. None of the pilot participants experienced problems understanding the statements on the questionnaire, thus no further changes were made before distributing the questionnaire.

To ensure that the minor changes made to items on the questionnaire did not negatively affect their reliability, we performed a Cronbach alpha coefficient test using SPSS version 25 (IBM Corp) to assess internal consistency among the items. The Cronbach alpha values of the constructs were as follows: trust propensity (.85), perceived security (.79), perceived privacy (.87), organizational reputation (.79), trust (.84), and intention to use wearable health devices (.77). Thus, changes to the wordings of items on our questionnaire did not have any negative impact on their internal consistency as the items were based on previously validated scales.

### Study Design

A cross-country electronic survey was conducted with participants from Kenya and South Africa. The questionnaire was implemented using Google Forms and distributed over a period of 4 weeks between September and October in 2018. To reduce the possibility of multiple responses by the same person, the Google Forms were set up to accept only one response per device. As potential participants were not offered any reward, there was little incentive for a participant to submit more than one response using multiple devices. The link to the Google Forms questionnaire was sent to a diverse group of undergraduate students pursuing Bachelor of Commerce, Engineering, Education, and Law degrees at 3 universities in Kenya (University of Nairobi, Kenyatta University, and Moi University) and 1 in South Africa (University of Pretoria) via email and WhatsApp, a social media platform. As acknowledged by Topolovec-Vranic and Natarajan [68], social media platforms such as Facebook, WhatsApp, LinkedIn, and Instagram provide new opportunities to recruit study participants. For example, the recruitment of potential study participants on social media platforms offers many benefits, including global reach, snowballing effect, and fast distribution [69]. To increase the participation rate, we requested participants to forward the link to the questionnaire to other potential participants. Responses to the questionnaire were automatically captured on the Google Forms.

Ethical approval for the study was granted by the Research Ethics Committee at the School of Information Technology, University of Pretoria. Participation in the study was completely voluntary; no identifying data were collected; and participants’ responses were anonymous. All respondents gave consent to participate in the study.

### Statistical Analysis Method

Data were analyzed using a mediation regression analysis and the between-group multivariate analysis of variance (MANOVA) test.

The mediation hypothesis analysis can be performed using the Monte Carlo CI, the Bayesian credible interval, the bootstrapping CI, or the Sobel methods [70]. However, the Monte Carlo CI, Bayesian credible interval, and bootstrapping CI tests have been shown to outperform the Sobel test [71-73].

To test hypotheses 1 to 5, we conducted a series of mediation regression analyses through bootstrapped CI using the PROCESS macro by Hayes [74]. PROCESS is a free, easy-to-use add-on for SPSS and statistical analysis system software. We used the PROCESS macro for SPSS to analyze our data. PROCESS comes with more than 70 predefined models [74]. We analyzed each of the 5 mediation hypotheses using the default model 4 in PROCESS. One of the benefits of the bootstrapped CI method for testing mediation is that it does not impose the assumption of a normal sample distribution. The method is also more robust when the sample size precludes the use of methods such as structural equation modeling [72].

H1 to H5 were tested using 1000 bootstrapped samples at 95% CI. To test H1 (the relationship between trust propensity and trust is mediated by organizational reputation), we ran the PROCESS macro with trust propensity as the independent variable, trust as the dependent variable, and organizational reputation as the mediating variable. Similarly, H2 (the relationship between trust propensity and the intention to use a wearable health device is mediated by trust) was tested by loading trust propensity as the independent variable, intention to use a wearable health device as the dependent variable, and trust as the mediating variable.

H3 (the relationship between organizational reputation and the intention to use a wearable health device is mediated by trust) was tested by loading organizational reputation as the independent variable, intention to use a wearable health device as the dependent variable, and trust as the mediating variable. We also tested H4 (the relationship between perceived security and the intention to use a wearable health device is mediated by trust) by loading perceived security as the independent variable, intention to use a wearable health device as the dependent variable, and trust as the mediating variable. Finally, H5 (the relationship between perceived privacy and the intention to use a wearable health device is mediated by trust) was tested by loading perceived privacy as the independent variable, intention to use a wearable health device as the dependent variable, and trust as the mediating variable.

The mediation regression analyses were carried out in 2 stages: (1) by loading all data from the 2 countries and (2) by separating the data according to participants’ country of residence. Separating the data enabled us to determine the extent of the differences in the mediation variables between the 2 cohorts.

To test H6 (there is a significant difference between Kenyans and South Africans in their intention to use wearable health devices), a one-way, between-group MANOVA test using SPSS was carried out to determine the factors that distinguish Kenyan and South African participants. The variables trust propensity, organizational reputation, trust, perceived security, perceived privacy, and intention to use a wearable health device were loaded as dependent variables, whereas country of residence was loaded as the independent variable.

To ensure that there is no serious violation of the underlying assumptions of MANOVA test, we tested for normality, linearity, univariate and multivariate outliers, homogeneity of variance-covariance, and multicollinearity. None of the underlying assumptions were violated, with the exception of the Levene test of equality of variances, where the variable, intention to use a wearable health device, has a significant value of *P*=.03. This value is slightly lower than the recommended value of *P*=.05.

We then used the Pillai trace to test for any statistically significant differences between the 2 groups. This is to ensure that the slight violation of equality of variance assumption did not influence the outcome of the MANOVA test. Pillai trace is known to be more robust than Wilks lambda when comparing groups in situations where there is some violation of the underlying assumptions of MANOVA [75].

## Results

### Demographics of Study Participants

A total of 232 responses were received. As shown in Table 1, there were 137 participants from Kenya and 95 from South Africa. A total of 58.2% (135/232) of participants were males, whereas 41.8% (97/232) were females. Within each country, 67.2% (92/137) of Kenyans were males, whereas 32.8% (45/137) were females. Gender distribution in South Africa was 45% (43/95) males and 55% (52/95) females.

### Correlation Between Research Constructs

The results of the correlation between the constructs are presented in Table 2. The constructs have weak-to-strong positive relationships with each other at *P*<.001. There is a weak positive relationship between trust propensity and the other 5 constructs (ie, perceived security, perceived privacy, organizational reputation, trust, and intention to use a wearable health device). The relationship between perceived security and the constructs perceived privacy, organizational reputation, trust, and intention to use a wearable health device is medium to strong, with the strongest relationship being between perceived security and perceived privacy (*r*=0.72; *P*<.001). Perceived privacy also has a moderate-to-strong positive relationship with the constructs perceived security, organizational reputation, trust, and intention to use a wearable health device, with the strongest being between perceived privacy and trust (*r*=0.72; *P*<.001). Between trust and the constructs trust propensity, perceived security, perceived privacy, organizational reputation, and intention to use a wearable health device, the strongest relationship is with organizational reputation (*r*=0.74; *P*<.001). The correlation between intention to use a wearable health device and the other 5 constructs is weak to moderate, with the strongest occurring between intention to use a wearable health device and trust (*r*=0.67; *P*<.001).

**Table 1:** Descriptive statistics of study participants.

Category and item			Frequency
**Gender, n (% per total sample)**			
	Male		135 (58.2)
	Female		97 (41.8)
**Gender per country, n (% within country)**			
	**Kenya**		
		Male	92 (67.2)
		Female	45 (32.8)
	**South Africa**		
		Male	43 (45)
		Female	52 (55)
**Country of residence, n (% per total sample)**			
	Kenya		137 (59.1)
	South Africa		95 (40.9)

**Table 2:** Pearson correlation (r) matrix for trust propensity, perceived security, perceived privacy, organizational reputation, trust, and intention to use a wearable health device at *P*<.001 (n=232).

Constructs	Trust propensity	Perceived security	Perceived privacy	Organizational reputation	Trust	Intention to use a wearable health device
Trust propensity	N/A^a^	0.398	0.359	0.325	0.382	0.258
Perceived security	0.398	N/A	0.720	0.494	0.598	0.478
Perceived privacy	0.359	0.720	N/A	0.620	0.721	0.542
Organizational reputation	0.325	0.494	0.620	N/A	0.742	0.620
Trust	0.382	0.598	0.721	0.742	N/A	0.671
Intention to use a wearable health device	0.258	0.478	0.542	0.620	0.671	N/A

^a^N/A: not applicable.

### Hypotheses Testing

The results obtained from testing H1, using data from both groups, show that the direct effect of trust propensity on trust is statistically insignificant at 0.11 (95% CI 0.048-0.177), as the CI of this direct effect includes 0 (Multimedia Appendix 3). In contrast, the indirect effect of organizational reputation is statistically significant at 0.22 (95% CI 0.143-0.309). We also split the data according to participants’ country of residence. This is to determine the differences in the mediating role of organizational reputation on the relationship between trust propensity and trust. The results show that organizational reputation has a statistically significant mediating role in the relationship between trust propensity and trust for both cohorts, albeit slightly lower in the case of the South African participants. These results support the hypothesis that the relationship between trust propensity and trust is mediated by organizational reputation.

The result of the regression analysis to test H2 is shown in Multimedia Appendix 4. The analysis reveals that there is no significant direct relationship between trust propensity and the intention to use a wearable health device with a direct effect of 0.001 (95% CI −0.056 to 0.058). When the indirect effect of trust is considered, the relationship between trust propensity and the intention to use a wearable health device becomes statistically significant at 0.26 (95% CI 0.172-0.349). Data from both countries were also analyzed separately. The results show that although trust plays a statistically significant mediating role in the relationship between trust propensity and the intention to use a wearable health device for the Kenyan participants, this is not the case for the South African cohorts. Hence, based on the combined results and the ones for Kenyan participants, the hypothesis that the relationship between trust propensity and the intention to use a wearable health device is mediated by trust is accepted.

Multimedia Appendix 5 illustrates the results of the mediation regression analysis to test H3 (the relationship between organizational reputation and the intention to use a wearable health device is mediated by trust). As shown in Multimedia Appendix 5, there is a statistically significant direct relationship between organizational reputation and the intention to use a wearable health device at 0.32 (95% CI 0.175-0.483). Although the mediating role of trust in the relationship between organizational reputation and the intention to use a wearable health device is also statistically significant at 0.35 (95% CI 0.218-0.494), this is not significantly different from the direct relationship between organizational reputation and the intention to use a wearable health device. A comparative analysis of data from both countries mirrors the combined result. Organizational reputation has a statistically significant direct relationship with the intention to use a wearable health device, with trust playing a slightly less indirect mediating role in the relationship for both cohorts. These results did not support H3. Thus, the hypothesis is rejected.

Multimedia Appendix 6 shows the results of the mediation regression analysis to test H4. The results show that there is no statistically significant relationship between perceived security and the intention to use a wearable health device with a direct effect of 0.07 (95% CI 0.000-0.147). This can be contrasted with the statistically significant indirect effect of trust at 0.36 (95% CI 0.255-0.461). A comparative analysis of data from both countries shows a similar pattern to the combined result. Thus, the results support our hypothesis that the relationship between perceived security and the intention to use a wearable health device is mediated by trust.

The results of the mediation regression analysis to test H5 are shown in Multimedia Appendix 7. Similar to the results of H4, there is no statistically significant relationship between perceived privacy and the intention to use a wearable health device with a direct effect of 0.07 (95% CI −0.009 to 0.150). The indirect effect of trust is however statistically significant at 0.42 (95% CI 0.282-0.557). The result of the comparative analysis of data from both countries is in line with the combined result. However, the result from Kenyan participants shows an indirect effect of 0.51 (95% CI 0.318-0.711). This is significantly higher than their South African counterparts where the indirect effect is 0.31 (95% CI 0.157-0.508). On the basis of the results, the hypothesis that the relationship between perceived privacy and the intention to use a wearable health device is mediated by trust is supported.

Table 3 provides a summary of the research hypotheses, the direct and indirect effects of the dependent and mediating variables for both countries and an individual country, and the hypotheses that are supported.

**Table 3:** Summary of research hypotheses findings.

Hypothesis	Mediation path	Direct effect (C’)			Indirect effect (ab)			Hypothesis supported
		All data^a^	Kenya	SA^b^	All data^a^	Kenya	SA^b^	
H1	Trust propensity - organizational reputation - trust	0.11	0.12	0.08	0.22	0.23	0.17	Yes
H2	Trust propensity - trust - intention to use a wearable health device	0.001	−0.04	0.04	0.26	0.29	0.26	Yes
H3	Organizational reputation - trust - intention to use a wearable health device	0.32	0.73	0.31	0.35	0.36	0.29	No
H4	Perceived security - trust - intention to use a wearable health device	0.07	0.04	0.09	0.36	0.43	0.25	Yes
H5	Perceived privacy - trust - intention to use a wearable health device	0.07	0.04	0.09	0.42	0.51	0.31	Yes

^a^All data: combined results.

^b^SA: South Africa.

As stated in the Methods section, we used the Pillai trace to test for any statistically significant difference between the Kenyan and South African cohorts because of the slight violation of the Levene test of equality of variances by the variable, intention to use a wearable health device. The MANOVA result suggests that there are statistically significant differences between Kenyans and South Africans in the combined dependent variables (*F*_6,225_=4.18; *P*=.001; Pillai trace=0.1; partial eta squared=0.1). We also considered the results of each dependent variable separately using a Bonferroni-adjusted alpha value of .008. There are statistically significant differences in the variables for both groups (Table 4). The only exception is in the organizational reputation variable, where there is no significant difference between the 2 cohorts. The mean ratings of the variables, as illustrated in Table 5, show that Kenyan participants’ ratings of the 6 variables are slightly higher than their South African counterparts. Table 5 also shows that despite the statistically significant differences between the 2 cohorts, the actual differences are less than 2 scales on average. The actual difference in the ratings for organizational reputation is less than 1 scale, which supports the results presented in Table 4.

**Table 4:** Multivariate analysis of variance results showing differences in variables between groups.

Variable	*F* value (*df*=1)	*P* value	Partial eta squared
Trust propensity	9.72	.002^a^	0.41
Perceived security	20.41	<.001^a^	0.82
Perceived privacy	13.47	<.001^a^	0.55
Trust	12.25	.001^a^	0.51
Organizational reputation	6.35	.01^b^	0.27
Intention to use a wearable health device	11.98	.001^a^	0.49

^a^Statistically significant difference between groups.

^b^No significant difference between groups.

**Table 5:** Participants’ mean score according to country of residence.

Variable and country of residence		Mean	SE	95% CI
**Trust propensity**				
	Kenya	13.179	0.415	12.362-13.996
	South Africa	11.496	0.345	10.816-12.177
**Perceived security**				
	Kenya	12.653	0.358	11.947-13.358
	South Africa	10.547	0.298	9.960-11.135
**Perceived privacy**				
	Kenya	11.947	0.388	11.183-12.712
	South Africa	10.095	0.323	9.458-10.731
**Trust**				
	Kenya	8.779	0.294	8.199-9.359
	South Africa	7.438	0.245	6.955-7.921
**Organizational reputation**				
	Kenya	5.747	0.181	5.390-6.104
	South Africa	5.153	0.151	4.856-5.451
**Intention to use a wearable health device**				
	Kenya	5.716	0.222	5.278-6.154
	South Africa	4.715	0.185	4.351-5.080

## Discussion

### Principal Findings

The results obtained from this study showed that organizational reputation has a significant direct influence on the trust in and intention to use a wearable health device. Study participants’ country of residence did not change the direct influence of organizational reputation on the trust in and intention to use a wearable health device. Similarly, trust has a significant mediating effect on the intention to use a wearable health device even in the presence of perceived security and perceived privacy. The results from this empirical study of 232 participants (Kenya, n=137; South Africa, n=97) using mediation regression analyses support hypotheses H1, H2, H4, and H5. The one-way, between-group MANOVA test also supports H6.

As hypothesized, organizational reputation has a statistically significant mediating effect on the relationship between trust propensity and trust. This mediating effect was present when data from the 2 countries were analyzed together and when we analyzed the data according to the country of residence. Similarly, as predicted, trust has a statistically significant mediating effect on the relationship between trust propensity and intention to use a wearable health device. This is the case for the combined data and Kenyan participants’ data. However, trust did not have a significant mediating effect on the relationship between trust propensity and the intention to use a wearable health device for South African participants.

Results from our study show that trust propensity on its own does not necessarily lead to the intention to use a wearable health device. Previous studies, such as those by Zhou [36], Colesca [37], and Lee and Turban [38], found that people’s trust propensity influences their trust in and intention to adopt new technologies. Findings from our study are in line with previous studies in this regard. However, this study goes beyond previous studies by suggesting that organizational reputation significantly mediates the relationship between trust propensity and trust. Our research shows that people with high trust propensity will be more likely to trust a wearable health device when the device’s manufacturer has a good reputation.

The hypothesis that trust plays a significant role in the relationship between organizational reputation and the intention to use a wearable health device is not supported by results from our study. Organizational reputation has a significant direct effect on the intention to use a wearable health device, with trust playing a lesser mediating role. Even when the country of residence is taken into account, organizational reputation on its own plays a significant direct role in the intention to use a wearable health device. Evidence from the literature suggests that good organizational reputation influences consumers’ trust and continued loyalty, irrespective of whether they live in a developed or developing country [47,50-52]. Thus, our study is in line with the ones from developed (eg, America and Spain [50,52]) and developing (eg, Iran and Nigeria [47,51]) countries. Our study also confirms the value of good organizational reputation.

Results from our study also show that perceived security and perceived privacy on their own do not significantly influence consumers’ intention to use wearable health devices, rather these factors are mediated by trust. Previous studies, such as those by Gu et al [28], Arpaci [76], and Damghanian et al [77], investigated perceived security and perceived privacy in relation to trust, not the intention to use new technologies. Our study extends these studies by presenting empirical evidence that the presence of perceived security and perceived privacy on their own do not have a significant influence on consumers’ intention to use a wearable health device. Rather, trust plays a significant mediating role in the intention to use a wearable health device.

Our assumption that there is a significant difference between Kenyans and South Africans in their overall intention to use wearable health devices is supported by the study results. There are statistically significant differences between the 2 groups on the variables trust propensity, perceived security, perceived privacy, trust, and intention to use a wearable health device. However, there is no significant difference between the 2 cohorts on the variable organizational reputation. The MANOVA results also confirm those from the mediation regression analyses, where organizational reputation is shown to have a significant direct influence on the intention to use a wearable health device for the 2 cohorts.

Although we did not delve deeper into the reasons for the differences in the factors that influence Kenyans and South Africans in their intention to use wearable health devices, the results are in line with our expectation. For instance, Morawczynski and Miscione [78] found that Kenyans are more trusting of the provider of M-PESA (mobile money) service (ie, the organization) due to its proven track record. They are, however, less trusting of individual agents that often act as intermediaries between customers and the service provider. Similarly, a study by the South African Human Sciences Research Council [79] found that South Africans generally have low levels of trust. However, in spite of their low level of trust, South Africans tend to demonstrate an increased level of loyalty to brands with a good image [80]. Hence, results from our study confirm what has been reported in the literature about the influence of positive organizational reputation on Kenyan and South African consumers.

### Study Contributions and Implications

The findings of this study made theoretical and methodological contributions to the field of uHealth. First, from a theoretical point of view, studies on the factors that influence the intention to use or adopt health technologies [28,30] did not consider the mediating effect of factors such as trust and organizational reputation. In contrast, we used a mediation regression analysis to provide deeper insight into the factors that mediate the relationship between a specific independent and dependent variable. Furthermore, our study is different from previous studies in that it is a cross-country study, whereas the previous studies are single-country studies. The cross-country nature of our study enriches the findings. Our study provides empirical evidence that organizational reputation has a significant mediating role in the relationship between trust propensity and trust. This study also confirms the fact that good organizational reputation has a positive influence on consumers’ trust in and intention to use wearable health technologies. Many of the studies on the relationship between organizational reputation and trust are predominantly about trust in web-based services [30,41,49,52]. Given the significant growth in the adoption of wearable health devices for monitoring PA, it is important to understand the influence that corporate image and trust have on consumers’ choice of wearable health devices.

Second, from a methodological point of view, our study uses an emerging approach for the mediation regression analysis, the PROCESS macro by Hayes (see the Methods section for some of the benefits of PROCESS) [74]. Our study demonstrates the effectiveness of the PROCESS macro by Hayes [74] and adds to the growing number of studies, such as those by Naidoo [81], Zhang et al [82], Huang et al [83], Barboza and Siller [84], Ahmed et al [85], and Supakong and Jarunratanakul [86], that uses the method.

From a practical perspective, our study has implications for the manufacturers of wearable health devices. Results from the study demonstrate the significant influence of organizational reputation and trust on consumers’ intention to use wearable health devices. In the absence of trust and good organizational reputation, factors such as trust propensity, perceived security, and perceived privacy may not necessarily drive consumers to adopt wearable health devices. A good reputation is an organizational asset [43-45]. Manufacturers of wearable health devices should capitalize on this asset to attract more potential adopters of wearable health devices.

### Limitations and Future Research

Although this study made theoretical, methodological, and practical contributions, it has limitations. For instance, we did not consider the moderating role of age on the factors that influence the intention to use wearable health devices. Furthermore, we did not explore the factors that could explain the differences in the 2 cohorts’ intention to use a wearable health device. Future studies can extend the research model by specifically investigating the roles of age and previous experience in the use of a wearable health device by using these variables as moderators to the factors that influence the intention to use a wearable health device. Such a study could also explore the potential effect of fitness tracker purchasing habits on the factors that influence the intention to use wearable health devices.

### Conclusions

In this study, we investigated the factors that influence consumers’ intention to use wearable health devices. More specifically, we considered the mediating roles of organizational reputation and trust in the intention to use a wearable health device. We collected data from 232 participants resident in Kenya and South Africa through a questionnaire implemented on Google Forms. A mediation regression analysis and MANOVA tests were used to analyze data. The study provides empirical evidence that organizational reputation has a significant mediating effect on the relationship between trust propensity and trust. In addition, we demonstrated that even when the country of residence is taken into account, organizational reputation on its own significantly influences consumers’ intention to use a wearable health device. Another important finding from the research is that perceived security and perceived privacy are not sufficient to motivate the use of wearable health devices. Trust is an important factor that drives this intention. The study provides deeper insight into the factors that influence the intention to use a wearable health device by investigating the factors that mediate this intention, as opposed to linear relationships between the factors. Without good organizational reputation and trust, factors such as trust propensity, perceived security, and perceived privacy have little influence on consumers’ intention to use a wearable health device.

## Appendix

Multimedia Appendix 1 Research model.

Multimedia Appendix 2 Research instrument.

Multimedia Appendix 3 Mediating role of organizational reputation in the relationship between trust propensity and trust, with regression coefficients, indirect effects, and bootstrapped CI.

Multimedia Appendix 4 Mediating role of trust in the relationship between trust propensity and intention to use wearable health devices, with regression coefficients, indirect effects and bootstrapped CI.

Multimedia Appendix 5 Mediating role of trust in the relationship between organization reputation and intention to use wearable health devices, with regression coefficients, indirect effects, and bootstrapped CI.

Multimedia Appendix 6 Mediating role of trust in the relationship between perceived security and intention to use wearable health devices, with regression coefficients, indirect effects and bootstrapped CI.

Multimedia Appendix 7 Mediating role of trust in the relationship between perceived privacy and intention to use wearable health devices, with regression coefficients, indirect effects and bootstrapped CI.

## Abbreviations

CWHD: consumer wearable health device
eCommerce: electronic commerce
eGovernment: electronic government
MANOVA: multivariate analysis of variance
NFC: near-field communication
PA: physical activity
uHealth: ubiquitous health
