# Supplementary figures and images for: The Mediating Role of Organizational Reputation and Trust in the Intention to Use Wearable Health Devices: Cross-Country Study
Source: JMIR Mhealth Uhealth. 2020 Jun 9;8(6):e16721. doi: 10.2196/16721 (PMC7312256; doi:10.2196/16721)

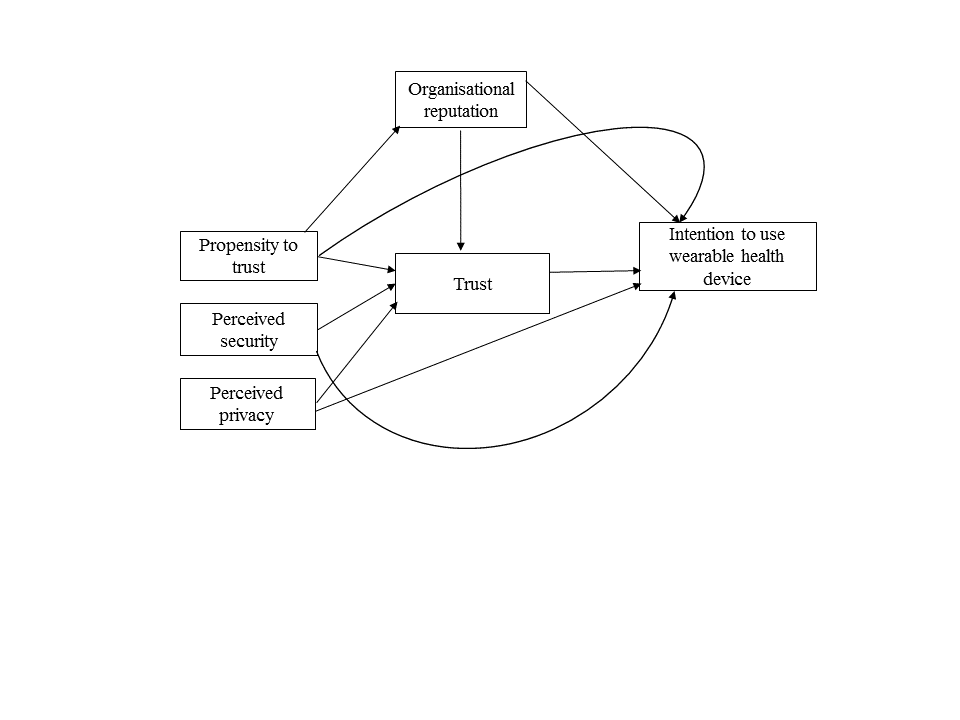

Supplement: Multimedia Appendix 1 [file mhealth_v8i6e16721_app1.png]

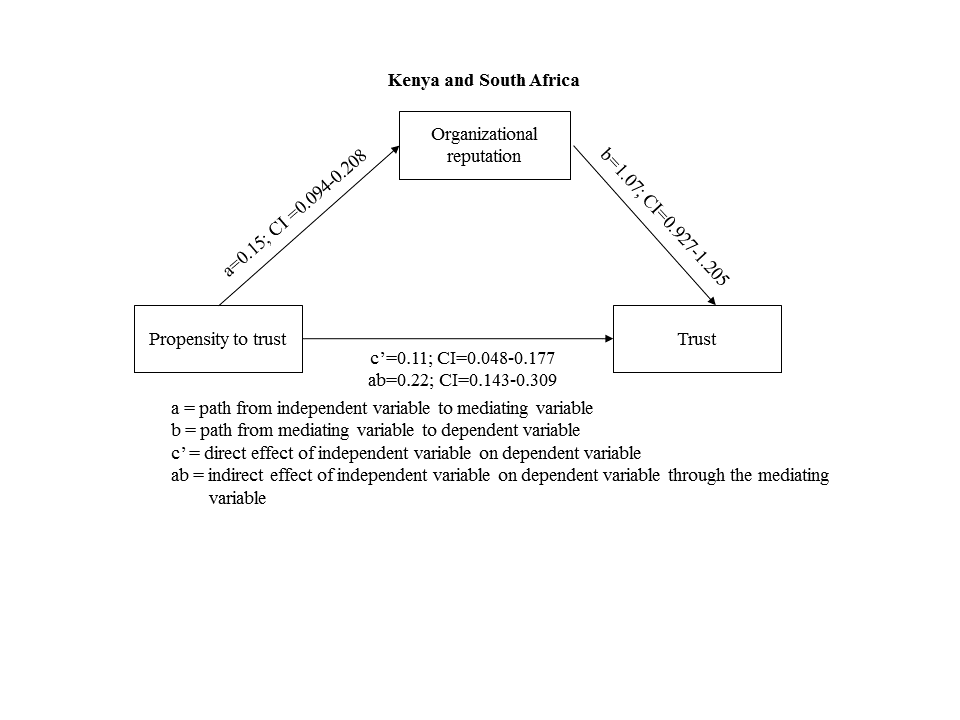

Supplement: Multimedia Appendix 3 [file mhealth_v8i6e16721_app3.png]

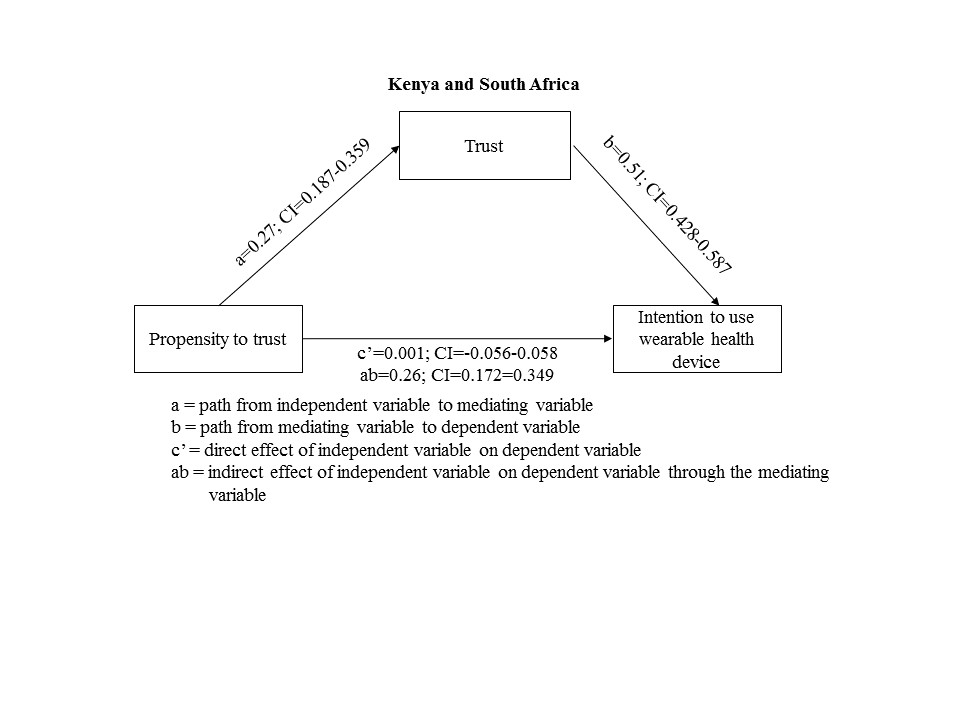

Supplement: Multimedia Appendix 4 [file mhealth_v8i6e16721_app4.png]

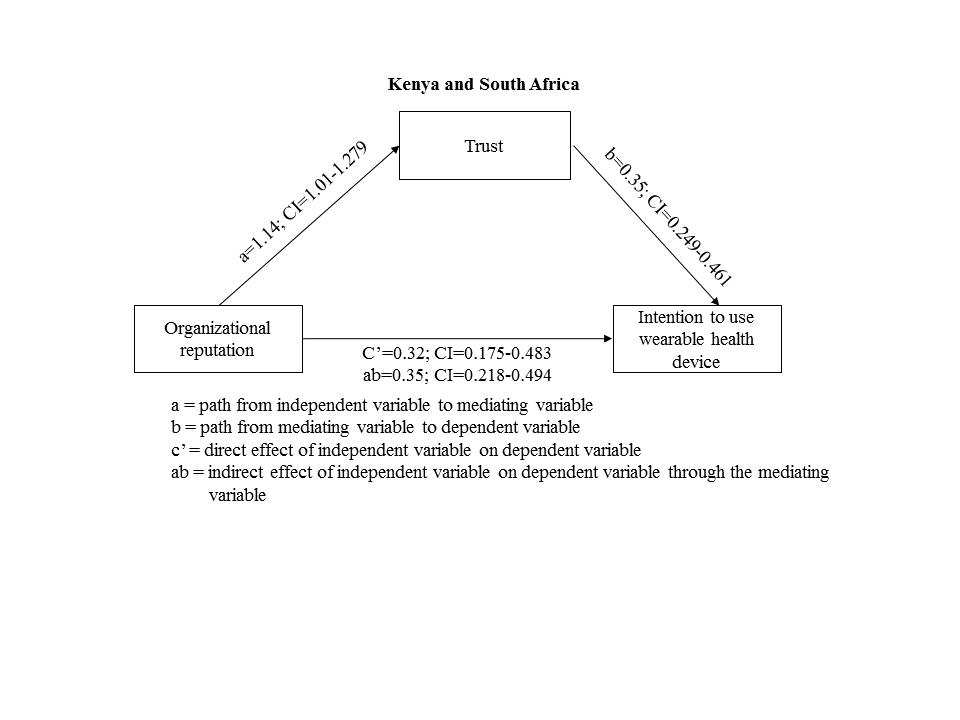

Supplement: Multimedia Appendix 5 [file mhealth_v8i6e16721_app5.png]

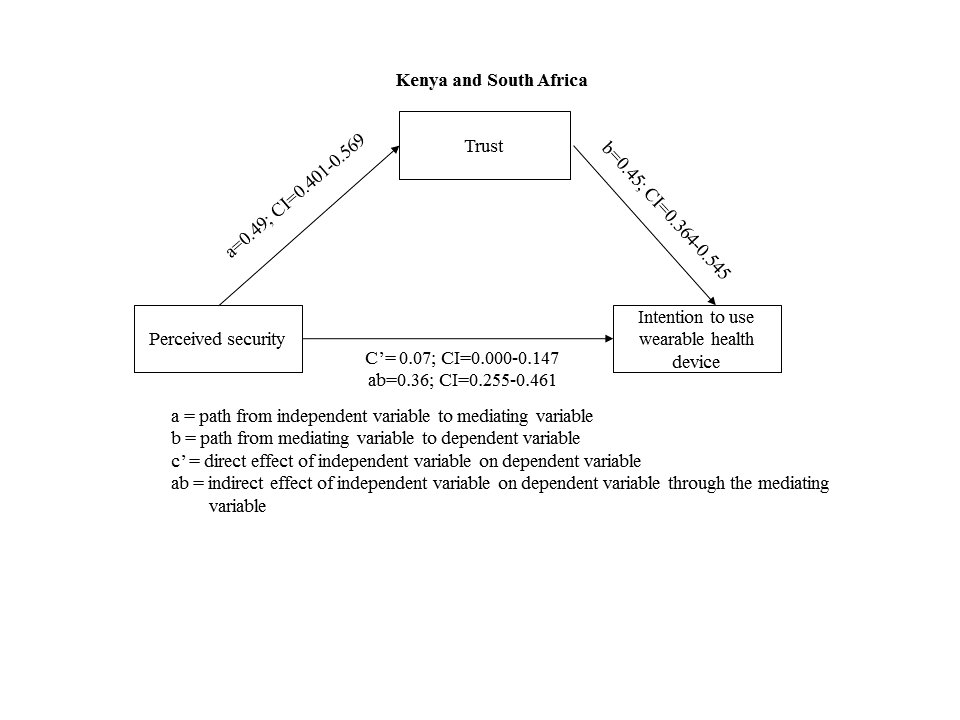

Supplement: Multimedia Appendix 6 [file mhealth_v8i6e16721_app6.png]

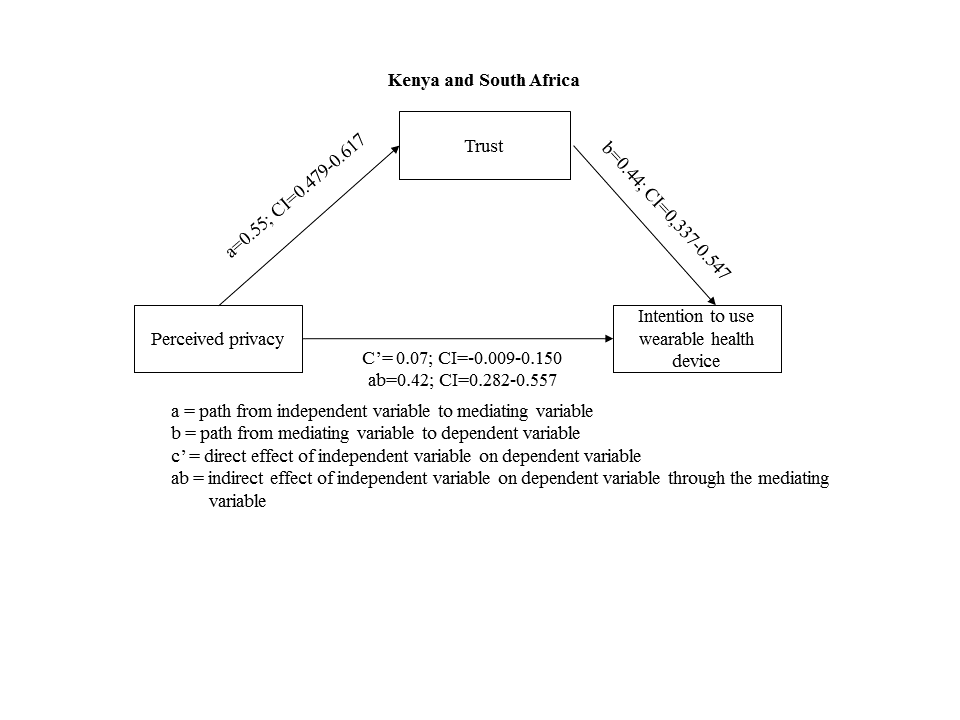

Supplement: Multimedia Appendix 7 [file mhealth_v8i6e16721_app7.png]
