# Supplementary material for: The Mediating Role of Organizational Reputation and Trust in the Intention to Use Wearable Health Devices: Cross-Country Study
Source: JMIR Mhealth Uhealth. 2020 Jun 9;8(6):e16721. doi: 10.2196/16721 (PMC7312256; doi:10.2196/16721)
Supplement: Multimedia Appendix 2 [file mhealth_v8i6e16721_app2.pdf]

Construct	Survey items	Cronbach alpha
Propensity to Trust (Cheung & Lee, 2001)	I easily trust a person or a thing<br>I tend to trust something / someone even though I have little knowledge about it<br>It is not hard for me to trust someone or something<br>My tendency to trust a person or thing is high	0.88
Perceived security (Flavián & Guinalíu, 2006)	I believe that wearable health devices have a way to ensure safe handling of my personal information<br>I believe that wearable health devices are designed to show great concern for the security of my personal information<br>I believe that wearable health devices cannot be hacked into by people with bad intention<br>I believe that wearable health devices are secure enough and the data they access, collect, and transmit will not be accessed or modified by third parties without my consent	0.95
Perceived privacy (Flavián & Guinalíu, 2006)	I feel safe when a wearable health device accesses my personal information<br>I believe that the wearable health device will not pass on my personal information to unauthorized third parties without my consent<br>I believe that the wearable health device respects my rights when accessing my personal information<br>I believe the wearable health device abides by personal data protection laws	0.92
Trust (Flavián, Guinalíu, & Torres, 2005)	I feel that I can trust wearable health devices<br>I believe in the information provided by the wearable health devices<br>I feel I can depend on wearable health devices to provide me with reliable information	0.86
Organizational reputation (Flavián et al., 2005)	I feel that the manufacturer of the wearable health device offers high quality products and services<br>I feel that the manufacturer of the wearable health device is concerned about its customers	0.76
Intention to use (Gefen, Karahanna, & Straub, 2003)	I am likely to use a wearable health device in the near future<br>I intend to buy a wearable health device in the future	0.83
